# Towards equity: a qualitative exploration of the implementation and impact of a digital educational intervention for pharmacy professionals in England

**DOI:** 10.1186/s12939-019-1069-0

**Published:** 2019-10-12

**Authors:** Asam Latif, Justin Waring, Kristian Pollock, Josie Solomon, Nargis Gulzar, Shahida Choudhary, Claire Anderson

**Affiliations:** 1 School of Health Sciences, Faculty of Medicine and Health Sciences, University of Nottingham, Nottingham, UK; 2 Health Services Management Centre, School of Social Policy, University of Birmingham, Birmingham, UK; 3 School of Health Sciences, Faculty of Medicine and Health Sciences, University of Nottingham, Nottingham, UK; 4 School of Pharmacy, University of Lincoln, Lincoln, UK; 5 Leicester School of Pharmacy, Faculty of Health and Life Sciences, De Montfort University, Hawthorn Building, Leicester, LE1 9BH UK; 6 School of Health Sciences, Faculty of Medicine and Health Sciences, University of Nottingham, Nottingham, UK; 7 Claire Anderson, Division of Pharmacy Practice and Policy, School of Pharmacy, University of Nottingham, Nottingham, UK

**Keywords:** Community pharmacy, Co-production, Digital learning, Medically under-served groups, Medicines use reviews (MURs), Normalisation process theory (NPT)

## Abstract

**Background:**

Patients belonging to marginalised (medically under-served) groups experience problems with medicines (i.e. non-adherence, side effects) and poorer health outcomes largely due to inequitable access to healthcare (arising from poor governance, cultural exclusion etc.). In order to promote service equity and outcomes for patients, the focus of this paper is to explore the implementation and impact of a new co-produced digital educational intervention on one National Health Service (NHS) funded community pharmacy medicines management service.

**Methods:**

Semi-structured interviews with a total of 32 participants. This included a purposive sample of 22 community pharmacy professionals, (16 pharmacists and 6 pharmacy support staff) all who offered the medicine management service. In order to obtain a fuller picture of the barriers to learning, five professionals who were unable to complete the learning were also included. Ten patients (from a marginalised group) who had received the service (as a result of the digital educational intervention) were also interviewed. Drawing on an interpretative analysis, Normalisation Process Theory (NPT) was used as a theoretical framework.

**Results:**

Three themes are explored. The first is how the digital learning intervention was implemented and applied. Despite being well received, pharmacists found it challenging completing and cascading the learning due to organisational constraints (e.g. lack of time, workload). Using the four NPT constructs (coherence, cognitive participation, collective action and reflexive monitoring) the second theme exposes the impact of the learning and the organisational process of ‘normalisation’. Professional reflective accounts revealed instances where inequitable access to health services were evident. Those completing the intervention felt more aware, capable and better equipped to engage with the needs of patients who were from a marginalised group. Operationally there was minimal structural change in service delivery constraining translation of learning to practice. The impact on patients, explored in our final theme, revealed that they experience significant disadvantage and problems with their medicines. The medication review was welcomed and the discussion with the pharmacist was helpful in addressing their medicine-related concerns.

**Conclusions:**

The co-produced digital educational intervention increases pharmacy professionals’ awareness and motivation to engage with marginalised groups. However structural barriers often hindered translation into practice. Patients reported significant health and medicine challenges that were going unnoticed. They welcomed the additional support the medication review offered. Policy makers and employers should better enable and facilitate ways for pharmacy professionals to better engage with marginalised groups. The impact of the educational intervention on patients’ health and medicines management could be substantial if supported and promoted effectively.

## Background

The right to physical and mental health without discrimination is recognised as a universal human right [1]. However, even within affluent societies, research indicates that vulnerable people from marginalised or ‘medically under-served groups’ (Table 1) experience significant inequitable access to health and find navigating health and screening services more challenging when compared to the general population [2–4]. Patients who identify as belonging to these groups encounter poorer patient-professional communication [5] and sometimes racism or cultural bias [6–8]. They also hold strong beliefs that they cannot be helped [9], face discrimination or disempowerment because of their circumstance [10], and are disenfranchised from mainstream primary health care [11]. Inequitable access to routine or preventative care risks higher rates of emergency admissions [12, 13] and increases pressure on acute services [14]. New strategies or care pathways to reduce inequitable healthcare access are urgently needed and are a priority for the NHS [15].

**Table 1:** Examples of communities or groups that could be medically under-served

• People with disability i.e., people with physical disability (e.g., a person in a wheelchair); people with visual impairment (Partially sighted/blind); people with hearing impairments (deaf) people with learning impairment (e.g., Downs syndrome, autism etc.) • People from Black, Asian and Minority Ethnic (BAME) communities • People who are homebound, from rural communities • People from Gypsy, Roma and Traveller (GRT) communities • People who are homeless or have no fixed address • People who are refugees or are seeking asylum • People from the lesbian, gay, bisexual and transgender, queer (LGBTQ) communities • People with mental health illness and stigmatised medical conditions (e.g., acquired immune deficiency syndrome (AIDS), epilepsy) • Older people, particularly with multiple morbidities and medicines • Young people (specifically men aged 18–25) • People from a low socio-economic status, long-term unemployed, low levels of health literacy • People with speech disorders (e.g., stutter) or language disorders e.g., from brain injury (stroke, dementia) • People experiencing substance misuse (e.g. alcohol, illicit drug dependency) • People who have experienced domestic/physical abuse • People who are sex workers • People in prison or those who are known to have been in prison	

Internationally, there is growing global interest in reforming primary care systems that seek to improve health equity [16]. Health educators have sought to reframe social determinants of health so they are seen less as “*facts to be known”* and more as “*conditions to be challenged and changed*” [17]. Included in this movement is health professional cultural competence training which has unfortunately shown limited impact beyond increasing knowledge and improving attitudes [18]. As such, there are now moves to teach the subject in a more nuanced way, moving away from pedagogic approaches of cultural competency towards a dynamic model that utilises frameworks of structural competency and critical consciousness [19, 20]. One limitation however, is that existing inequity reduction frameworks and models lack important guidance to organisations for the practical implementation of translating ambitions and macro policies into guided day-to-day action for frontline health professionals [21].

With this in mind, we draw on Normalisation Process Theory (NPT) to investigate the impact of a novel digital educational intervention (e-learning resource) to improve access in one NHS-funded community pharmacy service known as ‘Medicines Use Reviews’ (MURs). Co-production is an equal partnership (through the sharing of power) between service providers and service users (or other members of the community), where both parties make substantial resource contributions [22]. This concept has been shown to produce positive patient outcomes [23]. Moreover, on-line educational tools have also been shown to support professional learning and bring practice improvements [24, 25]. This study explores the implementation and impact of a novel co-produced digital educational intervention to promote service equity and outcomes for patients. Through this, we extend the debate on how difficult new patterns of behaviour are normalised in existing cultures of practices, processes and policies, as well as investigate the regulative and organisational elements that contribute to resistance [26].

### Medicine Use Reviews (MURs)

In light of the growing evidence suggesting patients experience significant problems taking medicines [27], concerns over medicine wastage, and adverse effects due to inappropriate polypharmacy [28], the United Kingdom (UK) Department of Health commissioned a national ‘Medicine Use Review’ (MUR) service in 2005. This was to be delivered from pharmacies as an optional ‘advanced service’ to support patient understanding and adherence to therapy [29]. The service is currently offered in approximately 90% of pharmacies and is free to patients. It is organised as an annual, one-to-one patient-pharmacist consultation aiming to resolve medicine adherence-related problems, address medicine-related concerns and to reduce avoidable waste. At the time, this move was based on emerging international evidence that pharmacist-led medication review models were feasible [30], and effective at improving medication adherence and health outcomes [31, 32]. There was also a willingness of the pharmacy profession to extend the pharmacists’ role beyond dispensing and information-giving, towards health promotion, prescribing and supporting medicine-use [33].

The implementation of MURs into pharmacist routines and practices however, has not been straight-forward. Questions have been raised over the variability in service delivery [34, 35]. With little formal monitoring or supervision, concerns have been expressed over the value to patients [36] and whether MURs are being targeted to “*local needs and patient priorities*” [37] leading to measures to phase out the service. One cause of these problems is the way pharmacies are remunerated for MURs. The NHS offers contractors a fee of £28 for each review performed, with the total number each pharmacy can claim subject to a cap of 400 annually. This cap, however, appears to have created a target-driven organisation culture [34, 35] in which contractors strive to claim as many reviews as allowed. There have been no requirements or incentives for pharmacists to recruit vulnerable patients from marginalised or medically under-served groups. With vulnerable patients from these groups likely to be in more need of support, there is scope for an intervention that raises awareness and engagement with people who may benefit the most. With over 3 million MURs conducted annually, we hypothesised that a co-produced digital educational intervention could be well placed to improve the provision of MURs to marginalised, medically under-served groups.

### Co-produced digital learning intervention

Novel, co-operative and inclusive initiatives that engage with vulnerable populations have been suggested as a way to improve healthcare access [9, 38]. This study used the co-production philosophy which acknowledges that stakeholders including services users and members of the public are best placed to advise on how services can be made more accessible to them, while also appreciating the input of front-line healthcare staff responsible for service delivery [22, 23]. In order to co-develop the educational intervention, mixed patient-professional workshops and qualitative one-to-one interviews with pharmacists and patients from under-served communities were undertaken. The final intervention comprised of three web-based digital learning resources:

1. Discovering and understanding under-served communities,
2. Exploring the medicine experiences and needs of patients who are under-served,
3. Effectively interacting and engaging patients who are under-served.

In order to engage the learner in interactive learning, each resource consists of a mixture of multimedia elements (i.e., audio, images, activities and illustrative videos) and represents approximately 15–20 min of learning activity. The first resource was co-developed following the revelation that there is low levels of knowledge among pharmacy professionals of who is ‘medically under-served’. The second sought to cultivate empathy and to better understand the lived experiences of such patients. The last resource, provided the learner with steps on how they could empower patients to take up the offer of an MUR. The resource is freely accessible online and can be accessed through our dedicated website found at:

https://www.nottingham.ac.uk/helmopen/rlos/pharmacy/practice/under-served/[39].

Further details of the development of the digital educational intervention have been reported in the study protocol [40] and elsewhere [41].

### Theoretical framework

#### Normalisation process theory (NPT)

Normalization process theory (NPT) is a widely used theoretical framework and was used in this study to identify, characterise and explain key mechanisms that facilitate or inhibit the embedding or normalisation of the complex intervention; it has been used widely to explore the implementation of new technologies or new processes in health care settings [42]. The theory proposes that “*material practices become routinely embedded in social contexts as the result of people working, individually and collectively, to implement them*” [43]. NPT offer a generalisable framework that can be applied across settings with opportunities for incremental knowledge gain over time [44]. One of the key strengths of the NPT is that it highlights potential social, cultural and organisational barriers to implementation, compared with more individualistic approaches such as the theory of planned behaviour [45]. A qualitative systematic review by McEvoy et al. [46] of studies applying the NPT framework to research implementation processes suggested healthcare researchers had positive responses and outcomes to the theory. In the pharmacy context NPT has been useful to examine experiences of delivering community pharmacy service to support adherence and self-management in chronic heart failure [47].

May and Finch [43] operationalise 4 constructs that form the framework:

1. Coherence (sense-making work)
  e.g. shared understanding
2. Cognitive participation (relational work)
  e.g. defining procedures
3. Collective action (operational work)
  e.g. allocation of work
4. Reflexive monitoring (appraisal work)
  e.g. determining effectiveness

## Method

This qualitative study is part of a larger appraisal of the co-produced digital educational intervention carried out between April 2016 and March 2019 [40]. The appraisal included a before / after survey study which was undertaken in community pharmacies located in Nottinghamshire, England. The embedded qualitative study aimed to develop an in-depth understanding of professionals’ situated practices, cultural context and organisational facilitators and constraints within which the digital educational intervention was implemented. All pharmacies in the Nottinghamshire area (*n* = 237) were approached. Pharmacy staff who were actively involved in the MUR service were invited to take part in the survey study. ‘Active involvement’ was defined as being involved with the process of identifying, inviting or undertaking MURs on a day-to-day basis. Survey responses were received from 122 pharmacies (involving 149 staff). At 3 months 62 participants had reported accessing and completing the e-learning.

This study draws on a total of 32 interviews including 22 pharmacy professionals (16 pharmacists and 6 dispensing staff) from the sample of pharmacy staff who agreed to participate in the survey study. Pharmacy professionals were purposefully selected reflecting variations in pharmacy ownership and employment role. In order to obtain a fuller picture of the barriers to learning, five professionals were selected who had been offered but unable to complete the digital educational intervention.

Ten patients were interviewed (who were identified as belonging to one or more medically under-served group(s)). Patients were recruited through 2 pharmacies by pharmacists who had completed the learning intervention. Once they had received their MUR from the pharmacist, the patient was then invited to the study. Patients and the recruiting pharmacy were offered a £25 High Street gift voucher as an inconvenience allowance. To avoid gift vouchers being used as incentives to take part in in an MUR, pharmacists were instructed to only invite patients to the study following their acceptance to have an MUR.

Qualitative, one-to-one, face-to-face semi-structured interviews were undertaken by AL. Patient / professional interviews lasted approximately 30–45 min and with the participants’ consent, were audio-recorded. Using NPT as a theoretical framework, we explored the impact of the e-learning on professional awareness, attitudes and behaviours engaging with and inviting people who were medically under-served. Professional motivation, barriers and facilitators to engage with the learning were also explored (see Table 2 in Appendix for topic guide). Patient interviews explored their health and illness, medicine-taking habits and experiences and personal value of the MUR. Their feelings about being approached for an MUR were also explored as well as thoughts on how healthcare professionals could better engage with, and improve services to, medically under-served groups (see Table 3 in Appendix for topic guide). Audio-recorded interviews were transcribed verbatim. Data were then imported into the qualitative analysis package NVivo [48] for coding using an interpretative analytical approach. The coding framework was developed through emerging themes as well as around the 4 NPT constructs (coherence, cognitive participation, collective action and reflexive monitoring).

## Results

### Implementation of the digital educational intervention

Our first theme considers how the resource was disseminated and the training undertaken by pharmacy professionals. Pharmacists were provided with an online link to the learning and were responsible for cascading this to relevant team members who were actively involved with the MUR service. The resource was described as concise, informative and relevant, accommodating a range of different learning styles. Despite its value, where there were short staffing issues, they were hesitant to share the learning with other staff over fears they would be burdening them with extra work. This hindered the translation of the learning into practice. Some professionals reported feeling guilty for being seen to prioritise learning over more pressing work; others reported completing the resource at home, in their own time:

*It’s extraordinarily difficult to find the time at work. There’s pressure to do 101 other things. I’m a travel health pharmacist, I do meningitis vaccines, I do flu vaccines, there’s a great pull on my time at work to do the actual training. (Pharmacist_Male_51yrs_Chain pharmacy).*

*We get a lot of e-learning and training here but myself I feel like I am always rushed to go through it as quickly as possible. … I feel I don’t want anyone to think I’m slacking, I feel I should be back on that counter. (Dispenser_Female_39yrs_Chain pharmacy).*

In a couple of cases, pharmacists were unable to engage or motivate their teams. In these cases undertaking the learning or sharing the resource was not assumed to be high on their list of priorities:

*Nobody else was interested in doing it I believe … I suspect it was a time element for them … (Pharmacist_Female_32yrs_Chain pharmacy).*

*I don’t know why we didn’t do it. It’s not something that was high on the list of things for them to do I guess … People who are old and been in the career for decades, change is hard. (Pharmacist_Male_46yrs_Independent pharmacy).*

### Impact of the learning: the process normalisation

Using the four NPT constructs, our second theme explores the process of normalisation and impact of the intervention on professional practice.

### Coherence

The coherence construct primarily focuses on the initial process of implementation and seeks to understand actor’s shared, or communal understanding of ideas, beliefs and meaning of any new intervention. For participants who had completed the learning, their awareness of medically under-served groups had improved. The learning opened their minds and allowed them to develop greater empathy with disadvantaged patients and their circumstances:

*So obviously these are real people giving their experiences of MURs and their struggles, it sort of puts you in their shoes, so you can understand where they are coming from. You can identify exactly how they feel and how they are being under-served*. *(Pharmacist_Male_44yrs_Independent pharmacy).*

As well as providing pharmacists opportunities for reflection, the resource also allowed scope for deeper consideration, introspection and for them to challenge personal unconscious biases:

*I don’t think anyone would maliciously try and go out and say “You know what, I’m going to avoid that definitely”, but I think you kind of look at it and subconsciously avoid it. I think now it’s probably opened my eyes that these are the kind of people who are more in need of this service. They are the people I should actively try and target. (Pharmacist_Male_29yrs_Independent pharmacy).*

*I didn’t even realise that I had a bias even … so the e-learning for me was brilliant … it challenged my assumptions, my biases, my awareness of cultures, and not a one size fits all kind of umbrella. (Pharmacist_Male_35yrs_Chain pharmacy).*

In a minority of cases, pharmacists were less empathetic in light of excessive work pressures and government cuts to their funding budgets. In these cases, given the infrequency with which these groups presented, it was easier for them to simply turn a ‘blind eye’:

*I don’t have the time to constantly search out vulnerable groups. Maybe that’s what we should be doing, but in the current climate, that’s an impossible thing to achieve. So, I’m going to go for the things that are contractually required of me to do because I’ve got enough on my plate already. (Pharmacist_Male_43yrs_Independent pharmacy).*

### Cognitive participation

Cognitive participation, refers to both the real and symbolic engagements and enrolments that must be made to make collective action possible [43]. In practice, this is the work, time and effort invested to integrate the new practice (effectively to buy into and support the new practice). The translation of the learning to make the MUR service more equitable was difficult; this was largely due to how the work of the MUR was organised. Most reported patient recruitment, even for those considered not to be marginalised, to be challenging. It was suggested that this was due to a general problem of patients not seeing the value of an MUR, or even understanding what was involved. With little patient-driven demand, organisational and personal strategies were deployed to facilitate the recruitment process. However, it became clear these micro-level decisions inadvertently disadvantaged people that were medically under-served. For example, stickers labelled ‘MUR’ were routinely attached to assembled prescriptions allowing staff to quickly identify eligible patients. However, patient invitations were circumvented where staff perceived it difficult to explain the service (i.e. where patient had English as a second language) or where patients were more challenging to recruit:

*Foreign people because there’s a bit of a language barrier, so it’s hard to explain it to them. And elderly, I guess, because they don’t really like to wait around, they are agitated … You can tell people who are a bit easier to approach than people who aren’t. (Dispenser_Female_36yrs_Independent pharmacy).*

Another example where certain patient groups were side-lined, was when pharmacists experienced heavy dispensing workloads. During these times, patients with fewer medicines for whom the MUR could be performed quickly and conveniently were preferred. They appreciated that patients who were ‘cherry-picked’ in this way may not receive as much benefit as for someone more vulnerable:

*Because of the time constraints most community pharmacists will take the easier option … It’ll be somebody who’ll be compos mentis, not deaf and not challenging, just to fulfil the numbers. Are they realistically the people who need the time and advice? (Pharmacist_Male_52yrs_Chain pharmacy).*

The main diver for offering the service was predominantly business-related. The fee for MURs were reportedly used as a means to offset or recoup ongoing government cuts to pharmacy budgets. Organisational pressure from management to recruit a targeted number of MURs was consequently evident. Role strain emerged where offering a professional service conflicted with notions of a target-driven commercial activity:

*You find yourself under some tension because it’s about the pressure to find one [MUR] and not the pressure to support the patient. (Pharmacist_Male_43yrs_Locum).*

*It is difficult sometimes to pick apart the need to hit the targets rather than whether or not it’s the most appropriate patient to do an MUR on. (Dispenser_Female_30yrs_Chain pharmacy).*

### Collective action

Collective action is the work that people do to enact or operationalise the new practice. This can be categorized into immediate (e.g. having the right skillset to perform the task) and organising factors (levels of support by the organisation). Regarding the skills to engage and recruit patients, practical issues around identifying and approaching people who were marginalised was still seen as a barrier. This applied, for example, where patients were unable to, or did not regularly attend the pharmacy. Identifying patients who were homeless was also perceived as difficult:

*The problem with this pharmacy is 70–80% of our business is delivery, sometimes we might not see the person for 6, 7 or 8 months. (Pharmacist_Male_40yrs_Independent pharmacy).*

*It’s very difficult to see if somebody’s homeless … In my six years I’ve never seen a prescription where it says “no fixed abode”, so are you telling me that in six years having served thousands of patients, if not a million patients, I’ve never served a homeless person? (Pharmacist_Male_29yrs_Independent pharmacy).*

Following the learning, there were few, if any, reported changes to the pharmacy’s operating procedures. While recognising that patients who were medically under-served could benefit to a greater extent compared to those not belonging to these groups, pharmacists still weighed up this potential value against whether they could afford the perceived extra time these MURs may take:

*If you had a choice of an easy patient and a not so easy one, what would you go for? That’s the reality and I’m sorry but that’s the basic truth. (Pharmacist_Female_59yrs_Independent pharmacy).*

### Reflexive monitoring

Reflexive monitoring is the construct which concerns itself with assessment of the efficacy of the new practice (appraisal work). This includes assessment of practice in a formal, communal sense, but also undertaking an informal individual appraisal. This ongoing assessment and how work is modified in response to these appraisals is important to maintaining ‘normalization’. Participants were asked to appraise their work following the learning. Overall, pharmacists recognised that recruiting patients from medically under-served groups was worthwhile. Despite the significant organisational constraints, several pharmacists had adjusted their practice, the learning instilling confidence for them to step out of their ‘comfort zone’ to adopt new procedures:

*I had a patient who was Chinese and somehow through some sort of signing or whatever I was able to get my message across. Maybe I wouldn’t have tried that MUR before, but I thought “no, I’m going to do this” … Today a patient who I never realised had ADHD got quite enraged in the pharmacy … Maybe before I may have banned him … He got quite aggressive over a refund … I said “let’s go and have a chat, let’s go and have a bit of time out”. That actually turned into an MUR, and it was as a result of the e-learning because of me thinking “under-served”. (Pharmacist_Male_35yrs_Chain pharmacy).*

*I would have been wary of doing an MUR with somebody who’s deaf, and since then I’ve done 2 people who are deaf … I’ve got somebody else who’s with them to come in and they helped with lip reading … It takes a bit longer, but actually I can see this type of group benefitting because nobody really takes the time to speak to them. (Pharmacist_Male_51yrs_Chain pharmacy).*

### Towards equity

To further appraise the impact of the learning intervention, our final theme explores the views of patients from medically under-served groups (who accepted an MUR from the pharmacist and then were subsequently interviewed). Most patients reported significant health issues and medicines-related problems. Several described discrimination; their social determinants of health acting as barriers to receiving health care:

*I was on heroin. People used to look at me and think “No not helping him”. I have been shunned from a few agencies. I’ve been shunned at hospital as well when they find out your background … They’ve made me feel it all, guilt. You’ve got yourself in that predicament … For years I didn’t go to the doctors … if I wanted medicine I could get it off the old boy down the street. I didn’t go and see the doctor because I was crucified for going in there. So, I ended up taking black market medication. (Patient_Male_44yrs_substance misuse).*

All patients agreed there was not enough health and medicine support for those who were vulnerable and medically under-served. There were several accounts of inadequately managed medicines, poor adherence and concerns over side-effects:

*I get confused as I’ve had a stroke … Sometimes, I know it sounds mad, I can’t be bothered because they’re too many … I’m blind as a bat so when reading them I’m not sure which ones I’m supposed to take, when I supposed to take or how I’m supposed to take it. (Patient_Female_58yrs_disability_homebound).*

When asked about their experience of the MUR, the ad hoc offer came as a surprise who were expecting just to collect their prescriptions. Being unfamiliar with this service, some were puzzled and even nervous as to why the pharmacist had asked to speak to them:

*Found it a bit strange … I thought you always had that chat with the doctor and thought the pharmacist was there to just sort out your medication. (Patient_Female_52yrs_mental health illness).*

*I felt quite nervous because at that time I didn’t realise why they were calling me in and I was chosen randomly, it felt quiet obscure. (Patient_22yr_ undertaking gender transition and registered disabled).*

During the consultation, all patients felt welcomed and were pleased by the way they were treated. They found the pharmacist to be kind and respectful:

*She explained about the medicine, “are you OK now, how are you feeling?” She was nice, very nice. (Patient_Male_55yrs_Black community).*

When asked to reflect on the purpose and value of the MUR, patients framed this as an opportunity for them to ask questions, learn and understand more about their medicines and how to take them more effectively. They appreciated the opportunity to sit with the pharmacist and have a chance to discuss their medicines:

*She told me I need to keep my blue inhaler with me at all times as I sounded chesty and wheezy and I have COPD. [Prior to that did you not keep your inhaler with you?] No, but will do now. (Patient_Male_44yrs_mental health illness).*

*She asked me if I wanted to ask any questions, but I didn’t feel like I had to, she covered all bases. She told me about my medications, told me when to take them, what I was to do if I took too many, an overdose, and things like that … I think it was to get me to understand what medication I was on and what they were for. (Patient_Female_39yrs_mental health illness).*

Although their MURs were able to resolve some problems, it was acknowledged that further help including ‘outreach services’ were urgently needed and that ‘government cut-backs’ to local services meant there were fewer resources to meet patient needs.

## Discussion

This study adds to the debate on how equitable improvements to primary care services for vulnerable populations can be promoted [49]. It explores the implementation of a new co-produced digital educational intervention to improve access, drawing in particular on NPT to understand how the intervention is enacted and embedded in practice. It also considers the impact of how the intervention and its implementation is experienced from the perspective of patients from the target population. Regarding the learning, pharmacists and their support staff reported several well documented barriers to learning. These were similar to those reported when undertaking continuing professional development (CPD) including not having time, lack of resources and interest [50]. Incorporating CPD as a form of in situ workplace learning has been suggested to improve engagement and professional practice [51]. However, our study suggests the perceived excessive workload within pharmacies, creates a barrier to undertaking new learning and constrains applying this knowledge in the workplace [52].

The findings from the four constructs of NPT framework revealed the complexity and extent to which the outcomes from the learning became normalised in practice. Under the coherence theme, the findings revealed there were improvements in awareness and better understanding of health inequities including how they occurred. However, cognitive participation and collective action remained limited due to organisational barriers and work place constraints. This hindered effective practice change to occur and to be sustained. When appraising their work (reflexive monitoring), most success was seen when pharmacists took it upon themselves to effect change; when this occurred, there was real potential for this to overcome the barriers to implementation and to achieve and maintain normalization. It could be that pharmacists who had been proactive, had positive attitudes to innovation or were, ‘early adopters’ [53]. Others have described these pharmacists as engaging fully with training and learning activities, being receptive to innovative behaviours and welcoming greater autonomy [54]. Nevertheless, effective ways to address inequity should seek to involve staff from all levels with equal motivation to participate in ‘readiness for change’ where these could contribute to improving practice [55].

Regarding the impact on patients, MURs were welcomed and the extra help and support these afforded were appreciated. In terms of Levesque et al. [56] model of conceptualising access, the learning had promoted abilities to perceive, seek, reach and engage with several medically under-served groups. It is well reported that people from these groups are more likely to manage health as a series of minor and major crises, rather than treating diseases as requiring maintenance and prevention [57]. Where the intervention was successfully implemented and professional learning was successfully implemented, there was potential for patient benefit. In times where questions are being asked about whether MURs represent value for money, targeting MURs to marginalised or medically under-served groups could be a valuable step towards demonstrating their relevance within certain medically under-served groups. However, it is clear that further research is needed to address the structural inequities within the system.

### Strengths and limitations

Whereas other studies using NPT have been criticised for not moving beyond a single stakeholder perspective [46], the present study used a range of stakeholders including patient, pharmacist and pharmacy support staff. These differing perspectives improved the credibility and transferability of the findings. Furthermore, in order to get a more balanced view, accounts were also taken from those who had not completed the digital educational resource. It is however not known what extent the digital learning accommodated for different learning preferences as this was beyond the scope of the present study.

Interventions can be applied at three levels: across the individual level that directly affect a patients’ social situation, at a health care organisational level aimed at professionals, and at a community or societal-level interventions, including social and political advocacy and research [58]. We accept that the intervention focuses predominantly on supply-side determinants to access, with less attention to facilitating demand-side determinants. As O’Donnell [59] notes, demand-side and supply-side barriers should be addressed concurrently in order to tackle the problem of access. Further research is needed to explore strategies on how awareness of the benefits of an MUR can be better promoted to patients from diverse backgrounds. In addition, more investment is needed to develop novel equity interventions [60] and evaluate their impact on patient care.

## Conclusions

Improving fairness, social justice and addressing inequitable access to health services features as a key priority for the UK NHS [15]. However, health disparities are still evident resulting in vulnerable patients experiencing significant problems with their health and managing medicines [41]. The co-produced digital educational intervention described in this paper aimed to support pharmacy teams to promote MURs to people medically under-served. Despite the significant challenges to implementing the learning, and normalising practice, patients from medically under-served backgrounds who were offered an MUR found this to be valuable and worthwhile.

## Data Availability

The datasets analysed during the current study are available from the corresponding author on request.

## Appendix

**Table 2:** Pharmacy staff topic guide

*Background*	
• Tell me a little about yourself and your work?	
• Tell me about your involvement in MURs (before the training) i.e. how do you identify / select patients for an MUR?	
• Are there any patients / groups you avoid?	
• What do you intend to achieve from undertaking the service?	
• *Before the training* what was your understanding of an under-served / or ‘hard-to-reach' group?	
*Evaluation of the use and implementation of the digital learning using the 4 NPT constructs*	
*Coherence*	
• Explore professionals’ description of the learning, meaning and sense making	
• Explore views of the purpose, benefits and value of the learning	
• Explore how the learning will fit with the overall goals and activity of the organisation	
*Cognitive Participation*	
• Explore professional opinions of whether the intervention is a good idea	
• Explore commitment and engagement with the learning	
• Explore professionals’ preparedness to invest time and energy	
*Collective Action*	
• Explore how the learning was cascaded to the pharmacy team	
• Explore the barriers and facilitators to effective translation into practice & compatibility with pharmacy standard operating procedures	
• Explore the impact on resources, responsibility between staff and other health professionals (e.g. GPs?)	
*Reflexive Monitoring*	
• Appraisal of the learning: has your knowledge of under-served communities changed as a result of using the e-learning, do you have any success stories?	
• Explore the effects of the learning on practice i.e. has this changed the way you undertake MURs as a result of the learning?	
• Feedback: In what ways could the e-learning be improved / adapted?	
Any final comments? / thank the pharmacy professional.	

**Table 3:** Patient topic guide

*Background & circumstance*	
• Tell me a little about your background? Prompt: work and lifestyle etc.	
• Explore health status, medical conditions and current concerns about health	
*Use of health services & medicine use*	
• Explore experiences of patients health care services	
• Do you have any concerns or problems with the medicines currently prescribed?	
• Explore adherence i.e. are medicine taken as prescribed or have these been changed?	
• Do you ever miss doses of your medicine, if so when / why?	
• Explore understanding of what medicines are for, concerns about side effects, perceptions of effectiveness, reluctance to take etc	
*Relationship with pharmacy/pharmacist*	
• How often do you make use of your local pharmacy and what are the reasons you use this?	
• What has your experience with pharmacies & their staff been (friendly, unfriendly, informative, discriminative etc.)?	
• Have you ever been offered a pharmacy consultation when you visit pharmacies? And what was your experience?	
• What sort of support are you aware of being available from your pharmacist? Can you recall any specific advice given by the pharmacist?	
• Have you ever asked a pharmacist for advice about your medicines (details)? Do you think the pharmacist is someone that you could approach for advice about your medicines? What prevents you from asking at the pharmacy?	
• If you have problems with your medication what if anything do you intend to do about it? What would you like to do?	
• How would you feel about being approached about using one or more of these additional services?	
*Specific questions relating to their marginalised or under-served status*	
• Explore self-identification / belonging to a medically under-served group	
• Explore communication / cultural challenges (people who have English as second language or from BAME community)	
• Can you describe the difficulties that your disability has in accessing or being offered community pharmacy services? (People with disability)	
• How do you think medicines services like the MUR can be better tailored to others in the same circumstances as you?	
*Questions about their experience of the MUR*	
• Explore awareness of the MUR and expectation of the service?	
• What did you think about the environment i.e. the size of the counselling room?	
• Can you describe how you felt during the consultation? (Prompt – friendly, comfortable, nervous?)	
• Explore patient’s perceived purpose of the MUR: What do you think the purpose of this service was?	
• What did you find most helpful & what did you find least helpful?	
• In what ways, if any, has the MUR helped you with your medicines? Have you changed the way you take your medicine according to this advice?	
• Are there any outstanding problems with your new medicine or any other medicine that you would like to have discussed with the pharmacist during the consultation?	
• Do you think the MUR was necessary for you?	
• Would you recommend this service to others?	
• Would you like to see any improvement in the way MURs are carried out?	
• Any final comments / thank the patient.	

## Abbreviations

MUR: Medicines Use Review
NPT: Normalisation Process Theory
